# Antifungal activity of 6-substituted amiloride and hexamethylene amiloride (HMA) analogs

**DOI:** 10.3389/fcimb.2023.1101568

**Published:** 2023-02-16

**Authors:** Kiem Vu, Benjamin J. Buckley, Richard S. Bujaroski, Eduardo Blumwald, Michael J. Kelso, Angie Gelli

**Affiliations:** ^1^ Department of Pharmacology, School of Medicine, University of California, Genome and Biomedical Sciences Facility, Davis, CA, United States; ^2^ Molecular Horizons and School of Chemistry and Molecular Bioscience, University of Wollongong, Wollongong, NSW, Australia; ^3^ Illawarra Health and Medical Research Institute, Wollongong, NSW, Australia; ^4^ Monash Institute of Pharmaceutical Science (ATMCF), Monash University, Parkville, VIC, Australia; ^5^ Department of Plant Sciences, PRB Building, University of California, Davis, CA, Australia

**Keywords:** amiloride, HMA, analogs, antifungal activity, *Cryptococcus neoformans*, MIC, MFC, *Candida* spp.

## Abstract

Fungal infections have become an increasing threat as a result of growing numbers of susceptible hosts and diminishing effectiveness of antifungal drugs due to multi-drug resistance. This reality underscores the need to develop novel drugs with unique mechanisms of action. We recently identified 5-(*N*,*N*-hexamethylene)amiloride (HMA), an inhibitor of human Na^+^/H^+^ exchanger isoform 1, as a promising scaffold for antifungal drug development. In this work, we carried out susceptibility testing of 45 6-substituted HMA and amiloride analogs against a panel of pathogenic fungi. A series of 6-(2-benzofuran)amiloride and HMA analogs that showed up to a 16-fold increase in activity against *Cryptococcus neoformans* were identified. Hits from these series showed broad-spectrum activity against both basidiomycete and ascomycete fungal pathogens, including multidrug-resistant clinical isolates.

## Introduction

Global estimates suggest that diseases caused by fungal pathogens affect over 1 billion people and kill approximately 1.7 million annually (Bongomin et al., 2017; Kainz et al., 2020). The severity of fungal diseases varies from asymptomatic in healthy hosts to disseminated, life-threatening infections in individuals that are immunosuppressed (Bongomin et al., 2017; Colombo et al., 2017). Over 90% of all reported fungal-related deaths are caused by *Cryptococcus*, *Candida*, *Aspergillus*, *Histoplasma* and *Pneumocystis* (Pfaller and Diekema, 2010). For the fungal species that are prevalent in the environment, such as *Cryptococcus, Histoplasma*, and *Coccidioides*, spores/desiccated yeast cells are inhaled and settle in the lungs where the infection can be asymptomatic to mild, but in susceptible hosts dissemination to other organs can result in death (Ellis and Pfeiffer, 1990; Woods, 2002; Eisenman et al., 2007; Brown et al., 2013).

The *Cryptococcus* spp. complex includes at least seven distinct species that can cause life-threatening disease and in countries where HIV infection is prevalent, cryptococcal meningitis is the most common form of adult meningitis (Zuger et al., 1986; Limper et al., 2017; Rajasingham et al., 2017). *Rhodotorula mucilagenosa*, a common environmental basidiomycete, is considered an emerging pathogen (Pfaller and Diekema, 2004; Wirth and Goldani, 2012). Most cases of *R. mucilagenosa* infections are bloodstream infections linked to central venous catheter use in susceptible hosts (Tuon et al., 2007; De Almeida et al., 2008; Wirth and Goldani, 2012; Falces-Romero et al., 2018; Kitazawa et al., 2018).

*Candida albicans* is the primary cause of 9.5% of all bloodstream infections in hospitals across the United States (Wisplinghoff et al., 2004). *Candida auris* was relatively unknown a decade ago but is today regarded as an emerging fungal pathogen that causes significant healthcare-associated outbreaks of bloodstream infections with high rates of mortality (Lockhart and Guarner, 2019). Although *Candida albicans* tends to be the most prevalent cause of candidiasis in humans, the last two decades has seen increases in infections caused by non-*C*. *albicans Candida* (NCAC) species. *C. glabrata*, *C. parapsilosis*, *C. tropicalis* and *C. krusei* are among the NCAC species that have emerged as important opportunistic fungal pathogens that are evolving to be more virulent and drug-resistant (Pfaller and Diekema, 2004; Wisplinghoff et al., 2004; Silva et al., 2012). Fluconazole-resistance among these *Candida* spp. is worrisome as fluconazole is the most commonly used antifungal agent for prophylaxis and treatment of *Candida* infections in resource-poor nations (Africa and Abrantes, 2016). Of particular concern is the high proportion of *C. auris* isolates that are resistant to three commonly used classes of antifungals: azoles, echinocandins and polyenes (Du et al., 2020; Frias-De-Leon et al., 2020). This multi-drug resistance creates significant challenges in clinical practice requiring the close monitoring of patients for treatment failure (Lockhart and Guarner, 2019; Hata et al., 2020).

Management of fungal diseases has become increasingly challenging due to the growing number of susceptible hosts and diminishing effectiveness of antifungal drugs. Indeed, the most pervasive and drug-resistant infections are now untreatable using first-line antifungals (Smith et al., 2015; Mpoza et al., 2018). This reality underscores the need to develop novel antifungal therapeutics with unique mechanisms of action able to effectively treat emerging resistant strains. While recent attempts at *de novo* antifungal drug discovery have produced only marginal success, drug repurposing (or re-positioning) provides an alternative approach to identify new indications for existing drugs (Kim et al., 2020; Wall and Lopez-Ribot, 2020).

In a recent study we examined whether amiloride, a K^+^-sparing diuretic, could be repurposed for the treatment of fungal infections (Vu et al., 2021). Amiloride, a WHO essential medicine, is a pyrazine acylguanidine originally developed as an inhibitor of renal epithelial Na^+^ channels (ENaCs) (Benos, 1982). We found that while amiloride has little antifungal activity, the 5-substituted analog, 5-(*N*,*N*-hexamethylene)amiloride, (HMA) shows modest minimum inhibitory concentrations (MICs) against isolates of *Cryptococcus* spp., and moderate synergy with several azole antifungals (Vu et al., 2021). Structure activity relationship (SAR) analysis revealed that hydrophobic substitutions on the 5-amino group of amiloride produced improvements in antifungal activity (Vu et al., 2021). HMA possesses nanomolar activity against Na^+^/H^+^ exchangers (NHEs) but minimal inhibitory activity toward ENaC, thus decreasing the clinical risk of ENaC-mediated hyperkalemia (Li et al., 1985; Kleyman and Cragoe, 1988). Collectively, our results suggested that HMA could serve as a starting point for antifungal drug development, where further optimization could produce new analogs with higher potency. Here, we investigated a library of 6-heteroaryl substituted HMA and amiloride analogs to determine whether further improvement in antifungal activity could be obtained from this scaffold. Compounds with substitutions at other positions around the pyrazine core of amiloride and HMA were also investigated.

## Material and methods

### Strains and media

KN99 is a common *Cryptococcus neoformans* serotype A laboratory strain derived from H99 (Nielsen et al., 2003). The *Candida* isolates and the *Rhodotorula* isolate were provided by Dr. G.R. Thompson, University of California, Davis. Drug-resistance of isolates was confirmed by the Fungus Testing Laboratory (San Antonia, Texas) and provided to us through Dr. G.M. Thompson. Strains were recovered from -80°C frozen stocks, grown in YPD (1% yeast extract, 2% bacto-peptone, and 2% dextrose) at 30°C and maintained on solid media containing 2% bacto-agar.

### Amiloride and HMA analogs

Amiloride.HCl was sourced from Sigma-Aldrich. Amiloride and HMA analogs were synthesized as previously described (Matthews et al., 2011; Buckley et al., 2018; Buckley et al., 2019).

### Antifungal activity testing by CLSI criteria

Susceptibility assays were carried out to determine MICs and MFCs according to the Clinical and Laboratory Standards Institute (CLSI). *In vitro* testing was carried out in RPMI 1640 medium containing *L*-glutamine, without sodium bicarbonate and buffered to pH 7.0 with MOPS in 96-well plates (96-well cell culture cluster, flat-bottom, Costar). Inoculum of *C. neoformans* (100 µL) was prepared in accordance with the CLSI standard (M27-A3), added to the 96-well plates and incubated for 48 h at 35 °C without shaking. Readings were taken by visual inspection of the opacity of wells. The minimum inhibitory concentration (MIC) was defined as the lowest drug concentration in a well at which 100% reduction in optical density was observed compared to the no-drug control well. The MIC was determined using concentrations from 2 µg/mL to 64 µg/mL. The minimum fungicidal concentrations (MFC) were determined by transferring the contents of the well identified as the MIC above and plated onto an YPD agar plate. The absence of colony forming units (CFUs) confirmed that the MFC was equivalent to the MIC.

## Statistical analysis

The MIC and MFC values reported in **Tables 1**, **2**, **S1**, **S2** are the result of at least 3 replicates.

**Table 1:** Antifungal activity of amiloride analogs against *Cryptococcus neoformans*.

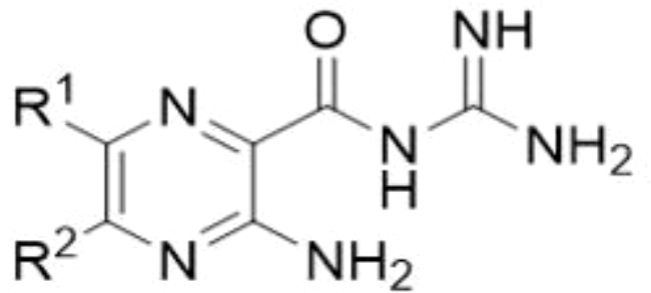							
		Cryptococcus neoformans				Cryptococcus neoformans	
R ^1^	Compound-R^2^	MIC	MFC	R ¹	Compound-R2	MIC	MFC
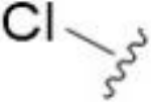	1 -NH_2_	64*	64*	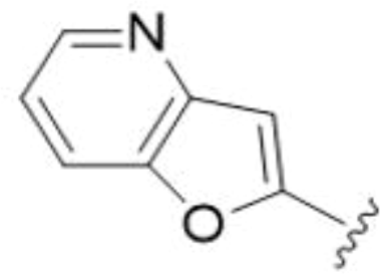	27 -N(CH_2_)_4_	>64	>64
	2 -N(CH_2_)_6_	64	64		28 -N(CH_2_)_6_	>64	>64
	3 -N(CH_2_)_2_O(CH_2_)_3_	>64	>64				
	4 -N(CH_2_CH_2_)_2_O	>64	>64				
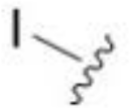	5 -NH_2_	>64	>64	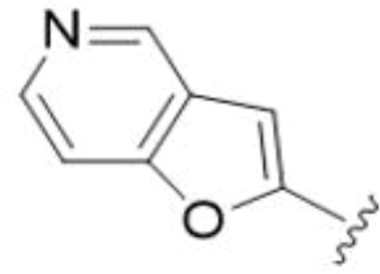	29 -NH_2_	64	64
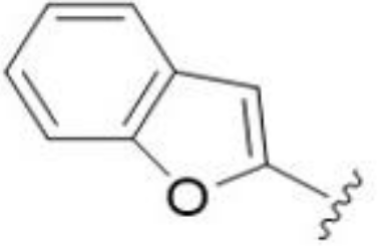	6 -NH_2_	64	64		30 -N(CH_2_)_6_	64	64
	7 -N(CH_2_)_4_	32	32	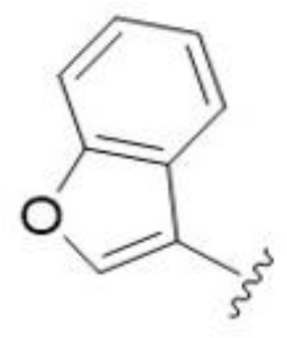	31 -N(CH_2_)_6_	32	32
	8 -N(CH_2_)_5_	16	16	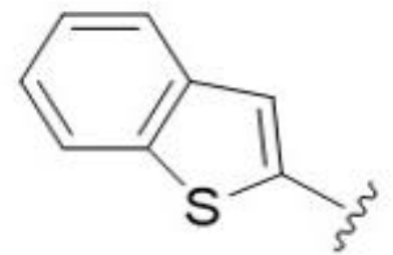	32 -NH_2_	>64	>64
	9 -N(CH_2_)_6_	16	16		33 -N(CH_2_)_6_	64	64
	10 -N(CH_2_)_2_O(CH_2_)_3_	>64	>64	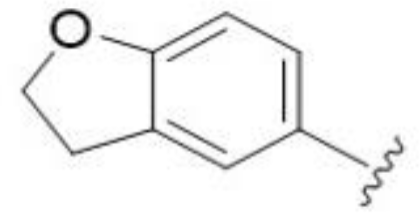	34 -N(CH_2_)_6_	32	32
	11 -NH(CH_2_)_2_Ph	8	8	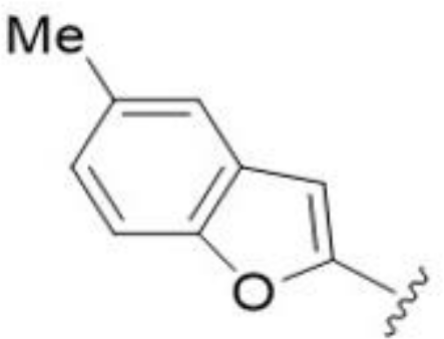	35 -N(CH_2_)_6_	>64	>64
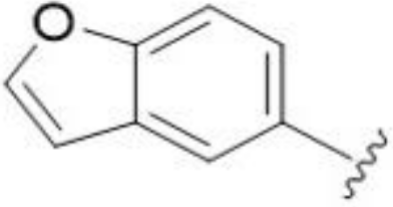	12 -NH_2_	>64	>64	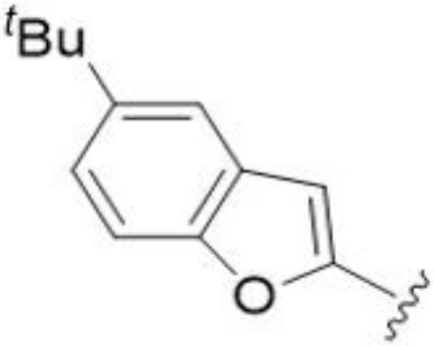	36 -N(CH_2_)_4_	>64	>64
	13 -N(CH_2_)_6_	16	16		37 -N(CH_2_)_6_	>64	>64
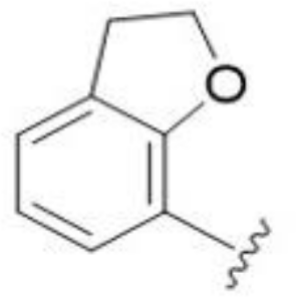	14 -NH_2_	8	8		38 -N(CH_2_CH_2_)_2_O	>64	>64
	15 -N(CH_2_)_6_	>64	>64		39 -N(CH_2_)_2_O(CH_2_)_3_	>64	>64
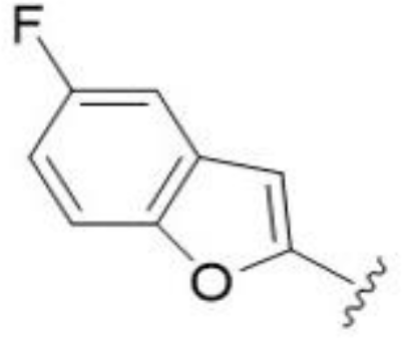	16 -NH_2_	4	4	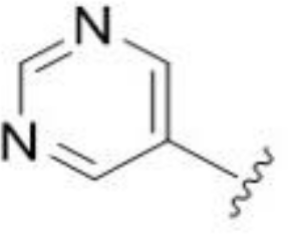	40 -NH_2_	>64	>64
	17 -N(CH_2_)_6_	4	8		41 -N(CH_2_)_4_	>64	>64
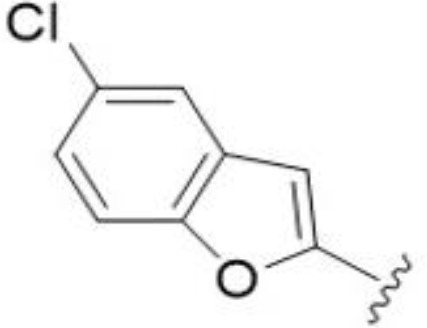	18 -NH_2_	>64	>64	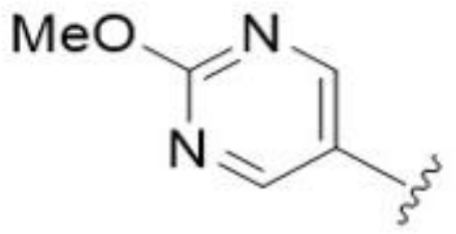	42 -NH_2_	>64	>64
	19 -N(CH_2_)_6_	16	16	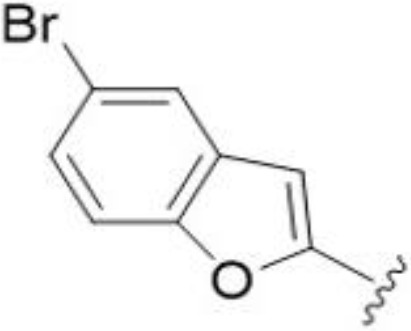	43 -NH_2_	>64	>64
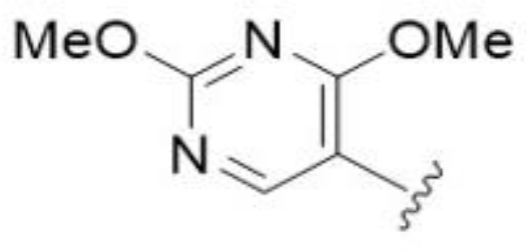	20 -NH_2_	32	32		44 -N(CH_2_)_6_	16	16
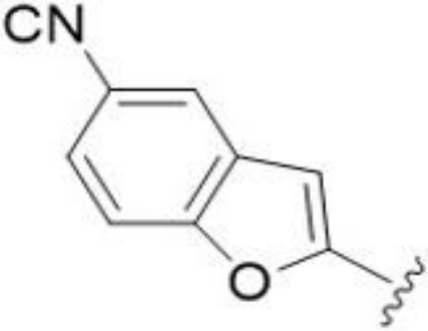	21 -N(CH_2_)_6_	8	8	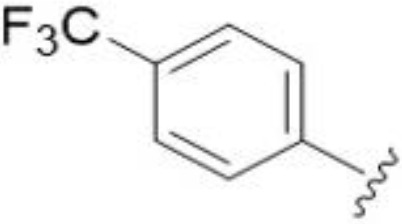	45 -NH_2_	>64	>64
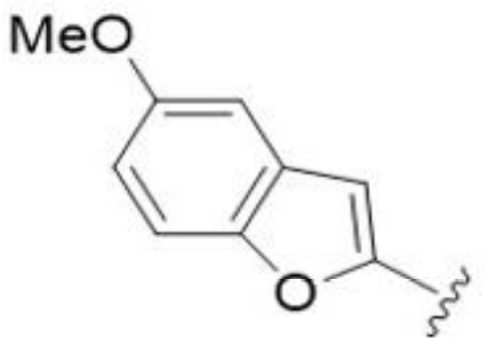	22 -N(CH_2_)_6_	16	32	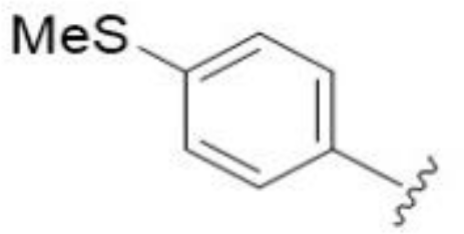	46 -NH_2_	>64	>64
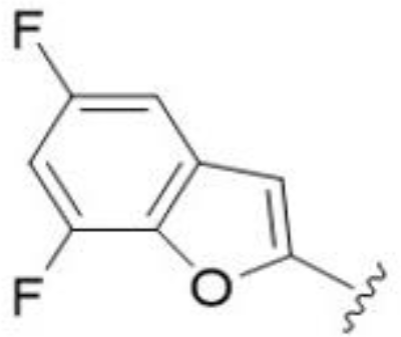	23 -NH_2_	8	8	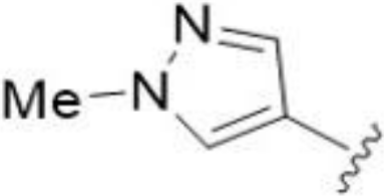	47 -NH_2_	>64	>64
	24 -N(CH_2_)_6_	64	64		48 -N(CH_2_)_6_	64	64
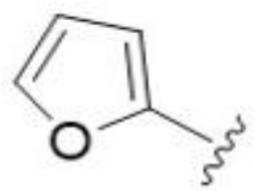	25 -NH_2_	>64	>64	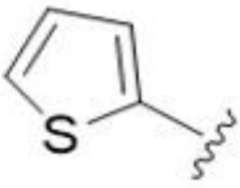	49 -NH_2_	>64	>64
	26 -N(CH_2_)_6_	16	16	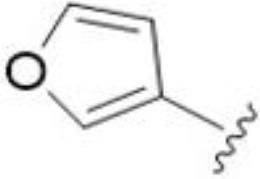	50-N(CH_2_)_6_	64	>64

MIC_100_, inhibitory concentration; MFC, minimum fungicidal concentration. All values represent µg/mL; *reported values for amiloride (Vu et al., 2021).

**Table 2:** Antifungal activity of amiloride and HMA analogs against *Candida* and *Rhodotorula* isolates.

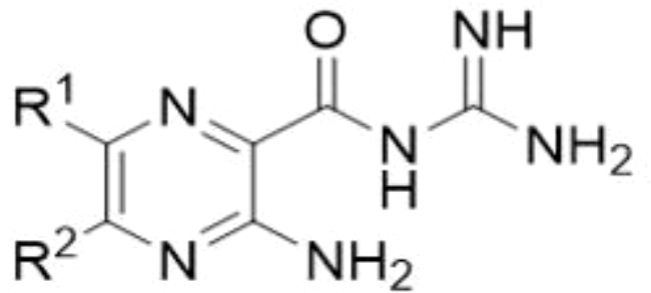									
		Rm*	Ca*	*Cg*	*C. auris*	Ch*	*Ck*	*Cp*	*Ct*
R^1^	Compound-R^2^	MIC/MFC	MIC/MFC	MIC/MFC	MIC/MFC	MIC/MFC	MIC/MFC	MIC/MFC	MIC/MFC
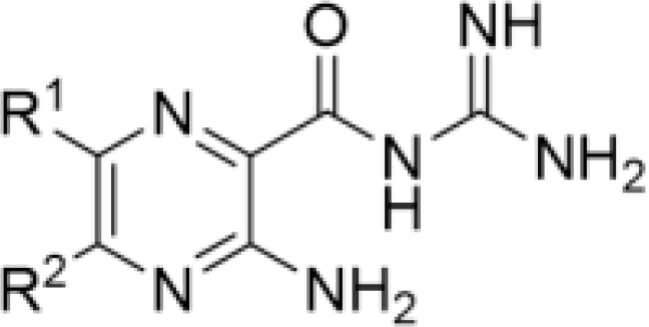	**2** -N(CH_2_)_6_	64/>64	>64/>64	64/>64	>64/>64	>64/>64	64/>64	64/64	>64/>64
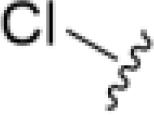	**8** -N(CH_2_)_5_	16/32	32/32	16/32	16/32	32/32	16/16	16/16	64/64
	**9** -N(CH_2_)_6_	16/32	32/32	16/32	32/32	16/16	16/16	16/32	64/64
	**11** -N(CH_2_)_2_Ph	8/>64	16/16	>64/>64	8/>64	8/8	8/8	8/8	8/8
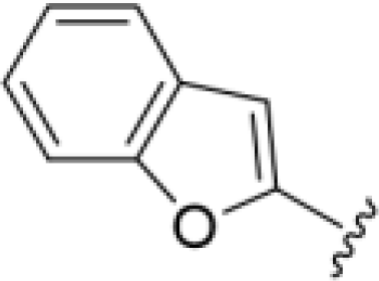	**13** -N(CH_2_)_6_	16/32	16/32	16/32	32/32	16/16	16/16	16/16	32/>64
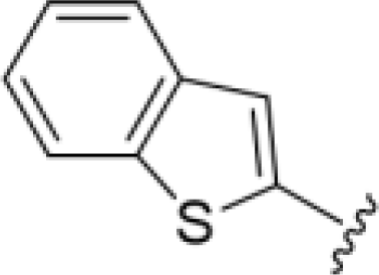	**14** -NH_2_	16/16	>64/>64	>64/>64	>64/>64	32/32	>64/>64	>64/>64	>64/>64
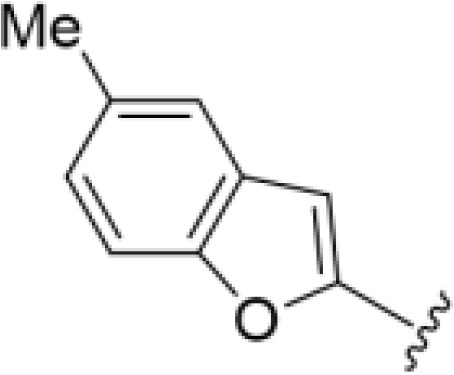	**16** -NH_2_	4/4	8/8	8/8	8/16	8/8	4/4	4/4	8/8
	**17** -N(CH_2_)_6_	4/4	4/4	4/4	4/4	4/4	4/4	4/4	<2/8
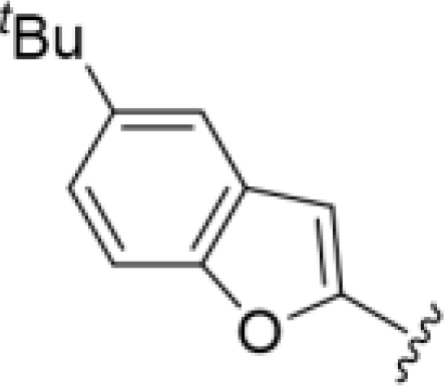	**19** -N(CH_2_)_6_	16/16	16/32	16/16	32/32	16/16	16/16	16/16	32/32
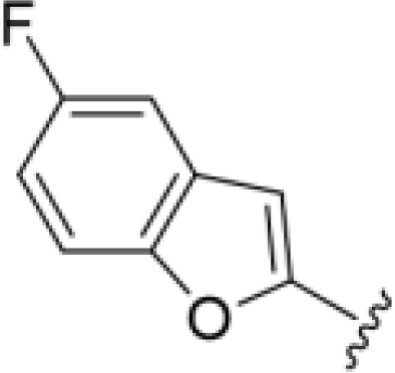	**21** -N(CH_2_)_6_	8/16	8/16	8/8	8/16	8/8	8/8	8/8	8/16
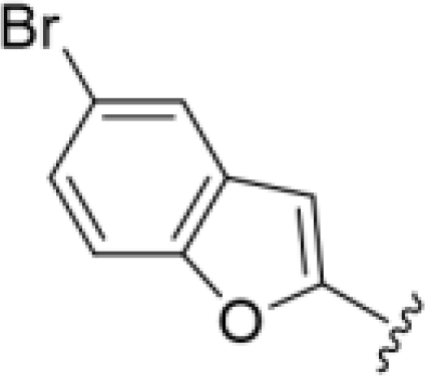	**22** -N(CH_2_)_6_	32/32	64/>64	64/64	64/>64	64/64	32/32	64/64	64/>64
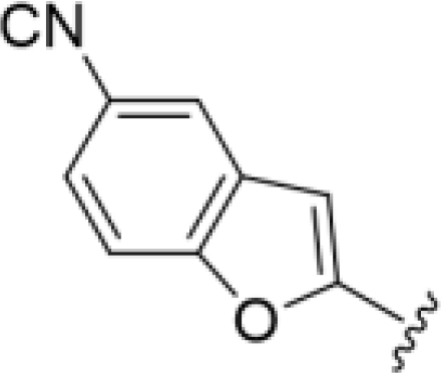	**23** -NH_2_	64/64	64/>64	64/>64	>64/>64	64/>64	32/32	16/16	>64/>64
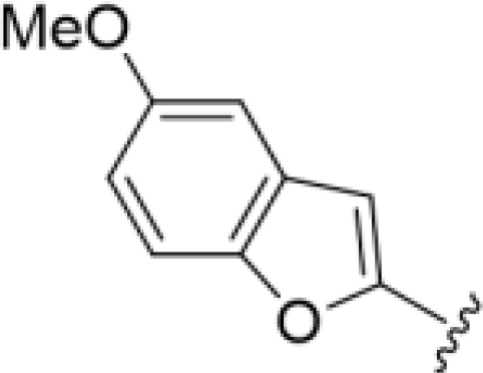	**26** -N(CH_2_)_6_	8/16	16/16	8/8	16/16	8/8	8/8	8/16	16/16
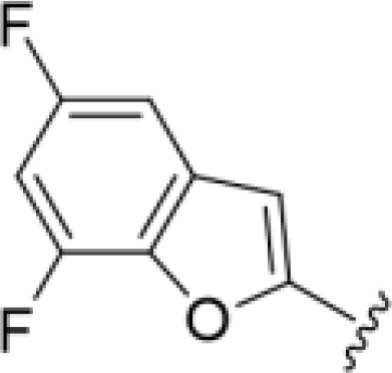	**44** -N(CH_2_)_6_	64/64	64/64	64/64	64/64	64/64	32/64	64/64	64/>64

Candida and Rhodotorula isolates. Rm, Rhodotorula mucilaginosa; Ca, Candida albicans; Cg, Candida glabrata; C. auris, Candida auris; Ch, Candida haemulonii; Ck, Candida krusei; Cp, Candida parapsilosis; Ct, Candida tropicalis. *denotes multidrug resistant clinical isolate. MIC_100_, minimum inhibitory concentration; MFC, minimum fungicidal concentration. All values represent µg/mL.

## Results and discussion

The antifungal activity of amiloride and HMA analogs carrying heteroaryl substitutions at the 5 and/or 6 position of the pyrazine ring were evaluated. Detailed physiochemical properties of HMA and 6-substituted match pairs have been reported in our recent work (Buckley et al., 2021a). A total of 64 analogs were examined by susceptibility assays against a strain of *Cryptococcus neoformans* (KN99) using the microbroth dilution method (**Tables 1**, **S1**). Screening of 45 6-(hetero)aryl substituted amiloride and HMA analogs reported previously revealed that antifungal activity was generally restricted to compounds bearing bicyclic heterocycles at the pyrazine 6-position (Buckley et al., 2018; Buckley et al., 2019; Buckley et al., 2021b; Hards et al., 2022). Consistent antifungal effects were seen for a series of 6-(2-benzofuran) analogs, with the HMA analog 9 and 5-piperidine 8 both showing MIC and MFC values of 16 µg/mL. Removal of the 5-azepane ring as in the matching amiloride analog 6 decreased activity, as did truncation of the amine at the 5-position, pyrrolidine 7, or incorporation of a polar O atom as in (1,4-oxazapane 10).

Substitution at the 5-azepane with a phenylethylamine 11 was favorable, producing a 2-fold increase in activity (MIC and MFC 8 µg/mL). Replacement of the ring O with S (2-benzothiophenes 12 and 13) did not improve activity. Introduction of a methyl substituent at the 5-position of the benzofuran ring increased activity by 8-fold (14 MIC and MFC 8 µg/mL) relative to the unsubstituted 2-benzofuran parent. Remarkably, this improvement was specific to the amiloride series, with no activity seen for the matching HMA analog 15 (MIC and MFC >64 µg/mL). 5-*^t^* Bu substitution produced the largest increase in activity in both series (16 and 17), lowering MIC and MFC by up to 16-fold (4 µg/mL). A drop in activity was seen for the 5-fluorinated amiloride analog 18, while no change was seen for the matching HMA analog 19. Larger halogens slightly increased activity, with 5-Cl 20 producing 2-fold lower MIC and MFC values for the amiloride analog (32 µg/mL) and 8-fold higher activity for 5-Br HMA analog 21 (8 µg/mL). This trend did not extend to 5-CN substitution, where no improvement in activity was seen with HMA analog 22. An 8-fold improvement was seen for 5-MeO amiloride 23 (MIC and MFC 8 µg/mL) while an 8-fold drop in activity was observed for the matching HMA analog 24 (MIC 64 µg/mL and MFC 64 µg/mL).

5,7-Difluorination as in amiloride 25 and HMA 26 did not improve activity for either series. Similarly, improvements were not seen for a series of 4-furopyridine 27 and 28 or 5-furopyridine analogs 29 and 30, indicating sensitivity to a polar N atom at these positions. Altering the connectivity of the benzofuran 31 and 34 or equivalent 2,3-dihydrobenzofurans 32, 33 and 35 did not improve activity. Furthermore, activity was poor or absent for a diverse selection of analogs bearing 5- and 6-membered (hetero)aryl groups at the pyrazine 6-position (36-43, 45-50), underscoring the necessity of the 6-(2-benzofuran) motif for antifungal activity.

One exception to this trend was seen for the 4-CF_3_phenyl HMA analog 44 (MIC and MFC 16 µg/mL), which showed equivalent activity to the 2-benzofuran HMA analog 9. In addition, no antifungal activity was seen for a separate series of amiloride analogs bearing a variety of secondary alkyl amines at the pyrazine 5-position (**Table S1**), in keeping with our earlier observations with 5-glycinyl analogs of amiloride (Matthews et al., 2011).

Further testing of 13 active analogs against the *Cn* isolate confirmed their antifungal and fungicidal activity (**Table S2**). 5-*^t^* Bu analogs 16 and 17 showed the highest activity against *Cn* (MIC and MFC 4 µg/mL. Phenylethylamine 11 and 5-Br benzofuran HMA analog 21 had average MICs of 7 µg/mL against *Cn* while the remaining 10 compounds displayed MICs ≥ 8 µg/mL.

Analogs with MICs and MFCs ≤ 16 µg/mL against *Cn* were examined against a panel of 7 *Candida* isolates, including multi-drug resistant *Candida auris* and *Candida haemulonii* strains, along with the drug-resistant basidiomycete isolate, *Rhodotorula mucilaginosa*. Susceptibility assays revealed that the 5-*^t^* Bu compounds 16 and 17, 5-Br benzofuran HMA 21 and 5,7-difluoro benzofuran HMA analog 26 were active against all fungal isolates, with MICs ranging from < 2 µg/mL to 16 µg/mL (**Table 2**). Phenylethylamine 11 inhibited growth of all isolates with the exception of *C. glabrata* (MICs ≤ 16 µg/mL), suggesting broad antifungal activity against both basidiomycetes and ascomycetes (**Table 2**). Broad spectrum activity was not seen for 4-CF_3_ phenyl analog 44, demonstrating the superiority of the 2-benzofuran group at the 6-position.

## Conclusion

We previously questioned whether HMA could elicit its antifungal effects *via* inhibition of the fungal homolog, the endosomal Na^+^/H^+^ exchanger Nhx1 (Vu et al., 2021). We found HMA to be similarly potent in *S. cerevisiae nhx1Δ* and *C. neoformans nhx1Δ* strains relative to wild type controls, suggesting Nhx1 inhibition is likely not responsible for antifungal activity (Vu et al., 2021). This conclusion was supported in this work by the absence of antifungal activity for 6-pyrimidine HMA analog 37, a compound reported as a nM inhibitor of human NHE1 (Buckley et al., 2021a). However, we cannot rule out potential inherent differences in activity/sensitivity of fungal and human Na^+^/H^+^ exchangers that could lead to differential effects of analog #37. The modest antifungal activity of HMA coupled with its poor stability *in vivo* preclude its advancement as a viable candidate for animal studies (Buckley et al., 2021a).

In summary, the 6-(2-benzofuran) class of amiloride and HMA analogs described here represent progress toward lead compounds suitable for further investigation. For example, HMA analog 9 showed 2 to 3-fold higher activity against a range of drug-resistant pathogenic fungi (MIC and MFCs 16-32 µg/mL). This analog does not show K^+^-sparing or diuretic activity and features a more favorable pharmacokinetic profile relative to HMA in mice and rat, supporting its future evaluation in animal models of fungal infection (Buckley et al., 2021a). Future studies will investigate synergy of these compounds with standard-of-care antifungals.

## Data availability statement

The raw data supporting the conclusions of this article will be made available by the authors, without undue reservation.

## Author contributions

KV performed the susceptibility testing. KV & BB performed data analysis. BB & RB & MK provided library of compounds. AG supervised study. KV, EB, BB and AG wrote the manuscript. All authors contributed to the article and approved the submitted version.

## Supplementary material

Supplementary Table 1 Antifungal activity of 5-substituted amiloride analogs against *Cryptococcus neoformans* isolates. MIC, minimum inhibitory concentration; MFC, minimum fungicidal concentration. All values represent µg/mL.

Supplementary Table 2 Susceptibility of *Cryptococcus neoformans* to 14 amiloride and HMA analogs. MIC, minimum inhibitory concentration; MFC, minimum fungicidal concentration. All values represent µg/mL.
