# Supplementary material for: Antifungal activity of 6-substituted amiloride and hexamethylene amiloride (HMA) analogs
Source: Front Cell Infect Microbiol. 2023 Feb 16;13:1101568. doi: 10.3389/fcimb.2023.1101568 (PMC10009331; doi:10.3389/fcimb.2023.1101568)
Supplement: Supplementary Table 1 — Antifungal activity of 5-substituted amiloride analogs against Cryptococcus neoformans isolates. MIC, minimum inhibitory concentration; MFC, minimum fungicidal concentration. All values represent µg/mL. [file Table_1.pdf]

Table S1. Antifungal activity of 5-substituted amiloride analogs against *Cryptococcus neoformans* isolates.

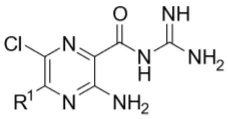

Compound	R^1	*Cryptococcus neoformans* MIC	*Cryptococcus neoformans* MFC
**51**	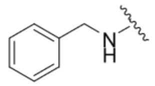	>64	>64
**52**	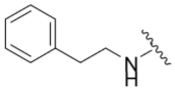	>64	>64
**53**	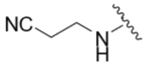	>64	>64
**54**	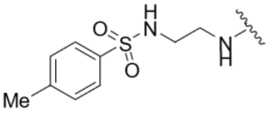	>64	>64
**55**	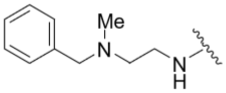	>64	>64
**56**	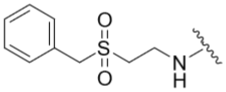	64	>64
**57**	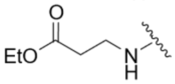	>64	>64
**58**	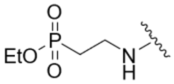	>64	>64
**59**	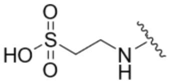	>64	>64
**60**	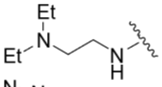	>64	>64
**61**	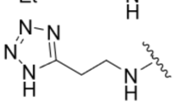	>64	>64
**62**	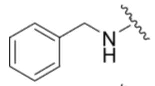	>64	>64
**63**	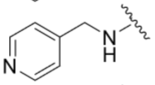	>64	>64
**64**	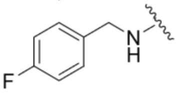	>64	>64

MIC_{100} = minimum inhibitory concentration; MFC = minimum fungicidal concentration. All values represent µg/mL.

Table S2. Susceptibility of *C. neoformans* (KN99) to 13 amiloride and HMA. MIC – minimum inhibitory concentration, µg/mL; MFC – minimum fungicidal concentration, µg/mL.

**Compound**	**Trial 1**		**Trial 2**		**Trial 3**	
#	**MIC**	**MFC**	**MIC**	**MFC**	**MIC**	**MFC**
**2**	64	64	64	64	64	64
**8**	16	16	16	16	16	16
**9**	16	16	16	16	16	16
**11**	8	8	8	8	4	8
**13**	16	16	16	16	16	16
**14**	8	8	8	16	8	8
**16**	4	4	4	4	4	4
**17**	4	8	4	4	4	4
**19**	16	16	16	16	8	8
**21**	8	8	8	8	4	4
**22**	16	32	16	16	16	32
**23**	8	8	16	16	16	16
**26**	16	16	16	16	8	8
**44**	16	16	16	16	16	16

MIC_{100} = minimum inhibitory concentration; MFC = minimum fungicidal concentration. All values represent µg/mL.
